# Fast and slow transient charging of Oxide Semiconductor Transistors

**DOI:** 10.1038/s41598-017-12155-3

**Published:** 2017-09-19

**Authors:** Taeho Kim, Sungho Park, Sanghun Jeon

**Affiliations:** 1 Department of Applied Physics, Korea University, 2511, Sejongro, Sejong, 446-712 Republic of Korea; 2 Department of Chemistry, Daejin University, Sundan-dong, Phochon-si, Gyeonggi-do 487-711 Republic of Korea

## Abstract

The comprehension of the governing mechanism which affects device instability is one of the most important requirements for the formation of reliable oxide-thin film transistors (TFTs). However, a quantitative analysis of the dominant mechanism of device instability, which stems from charge trapping induced by defects at the oxide semiconductor interface as well as in its bulk, has not yet been systematically performed. In this study, we examined subgap states, charge-transport dynamics, and various trap characteristics of oxide TFTs by multi-frequency *C*–*V*, pulse *I*–*V*, and transient current methods to achieve a comprehensive understanding of carrier transport and charge trapping mechanisms. We found that the charge trapping behavior of the tested amorphous InHfZnO (*a*-IHZO) TFT follows a multi-trapping mechanism, such as temperature-independent fast transient charge trapping by resonant drift of the injected electron and temperature-dependent slow transient charge trapping by charge transport from occupied to unoccupied traps. Understanding fast charging and slow charging described in this study can help to understand the root cause of device instability of oxide TFTs and ultimately improve stability and reliability characteristics.

## Introduction

In recent times, amorphous oxide semiconductor-based TFTs have been attracting enormous attention for display applications^1–5^, owing to their steep subthreshold slope (~0.2 V/decade), high field-effect mobility (5–100 cm^2^/eV·s), and low-temperature fabrication process^6–8^. High field-effect mobility enables fast switching, which is especially important for display technology^9,10^. In this research area, various amorphous-oxide semiconductor materials, such as InGaZnO, InHfZnO and InSnZnO have been studied to secure high stability and high reliability while maintaining high mobility^11–17^. However, the device instability and reliability issues of amorphous oxide semiconductor-based TFTs remain as potential issues faced by their production^18–23^. Interfacial and bulk defects in amorphous-oxide semiconductors result in significant charge trapping effects, leading to device instability and reliability issues. Therefore, an accurate understanding and precise management of defects in oxide semiconductors are required for the success of oxide TFTs^24–29^. The study of the fast and slow charge trapping were reported by many groups with various methods. Ramon *et al*., reported that the fast and slow charge trapping were investigated using the charge pumping technique^30^. U. Jung *et al*., suggested that the quantitative estimation of defects contributing to charge trapping using the discharge current analysis method^31^. These previous studies suggested that charge trapping is affected by both shallow (interface) and deep state (bulk) defects. Despite these studies, however, possible origins and information about locations of charge trapping remains unclear.

In our work, we investigated fast and slow transient charging behaviors in oxide TFTs and their effects on electrical characteristics. To this end, we employed a multi-frequency measurement (MFM) technique to evaluate the subgap density of states (DOS)^32^ as well as pulse *I*–*V* (PIV) and transient current methods to study time-dependent charge trapping phenomena^33–42^. In addition, we consider a model for electron charging behavior of oxide TFTs utilizing measurements of transient current with temperature. This shows that transient charge trapping follows two different type processes such as a fast electron charging process where electrons are injected into shallow defects (fast transient charging) and thermally activated electron migration via trap-to-trap conduction (slow transient charging). The former is responsible for mobility degradation and *V*_*TH*_ instability, while the latter is responsible for long-time stress *V*_*TH*_ instability. As a result of this study, it was found that DOS through MFM measurement technique was exponentially distributed in the shallow energy state region, and fast & slow charge trapping occurred relatively shallow energy in range of less than 0.4 eV below the conduction band minimum through short and long pulse measurement technique. This model enables comprehension of the dynamic charge trapping behavior of oxide TFTs^43^.

## Results

**Figure 1:**
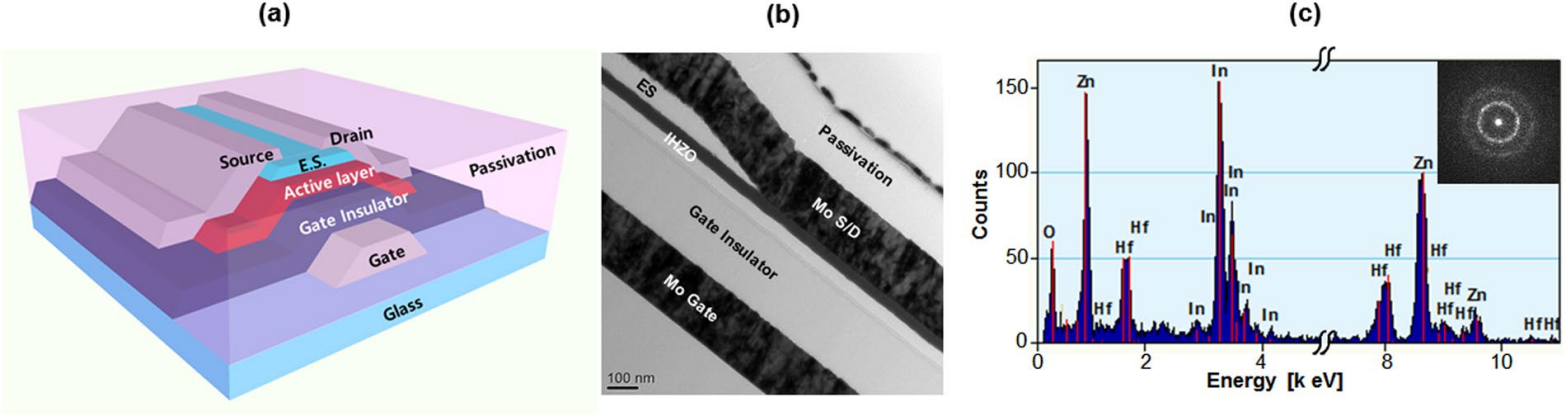
(**a**) Schematic structure and (**b**) cross sectional transmission electron microscopy image of an *a*-IHZO TFT. (**c**) Energy dispersive spectroscopy data of an *a*-IHZO TFT. The inset shows a transmission electron diffraction pattern.

**Figure 2:**
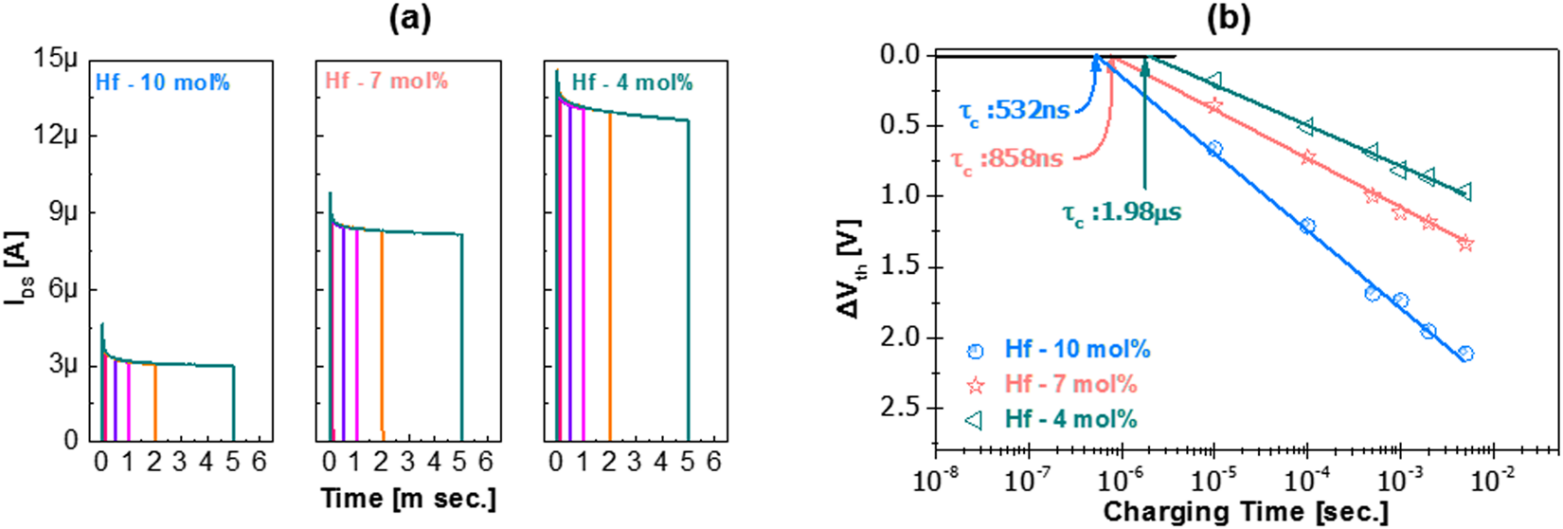
(**a**) Transient current versus charging time curves and (**b**) distributions of threshold voltage shift with charging time with various Hf contents.

Inverse staggered TFTs with *a*-IHZO semiconductor and molybdenum metal electrodes were fabricated. Schematics of a fabricated oxide TFT and a high annular transmission electron microscopy image are presented in Fig. 1(a) and (b), respectively. The semiconductor material composition was verified by analyzing the energy dispersion spectrometry, shown in Fig. 1(c) and the structure was confirmed by a transmission diffraction pattern (see the inset of Fig. 1(c)). After the fabrication of the a-IHZO TFTs, in order to confirm the influence of the Hf content on the IHZO films, the transient current versus charging time curves (the charging time range is from 5 µs to 5 ms.) were measured with various Hf contents, as shown in Fig. 2(a). The charge trapping effect causes a reduction in the drain current with time. The threshold voltage shift (ΔV_th_) was determined from the transient current versus charging time curves by using the equation, ΔV_th = _ΔI_DS_·(V_GS_ − V_th_)/I_DS_. Where ΔI_DS_ is difference of the drain current during the pulse width (charging time), I_DS_ is the maximum value of the drain current before the pulse width, V_GS_ is the pulse amplitude, and V_th_ is the threshold voltage. From this equation, distributions of ΔV_th_ were plotted with respect to charging time, as shown in Fig. 2(b). Then, the critical charge trapping time (t_c_) was determined. We found that t_c_ increased with decreasing Hf content in the *a*-IHZO (t_c_ values of 4, 7, and 10 mol% Hf content in the *a*-IHZO TFT are 1.98 μs, 858 ns, and 509 ns, respectively). Therefore, the Hf content in a-IHZO films obviously influences charge trapping mechanism. This experimental results showed that the incorporation of high Hf content in a-IHZO semiconductor increased the defect density, resulting in device instability.

**Figure 3:**
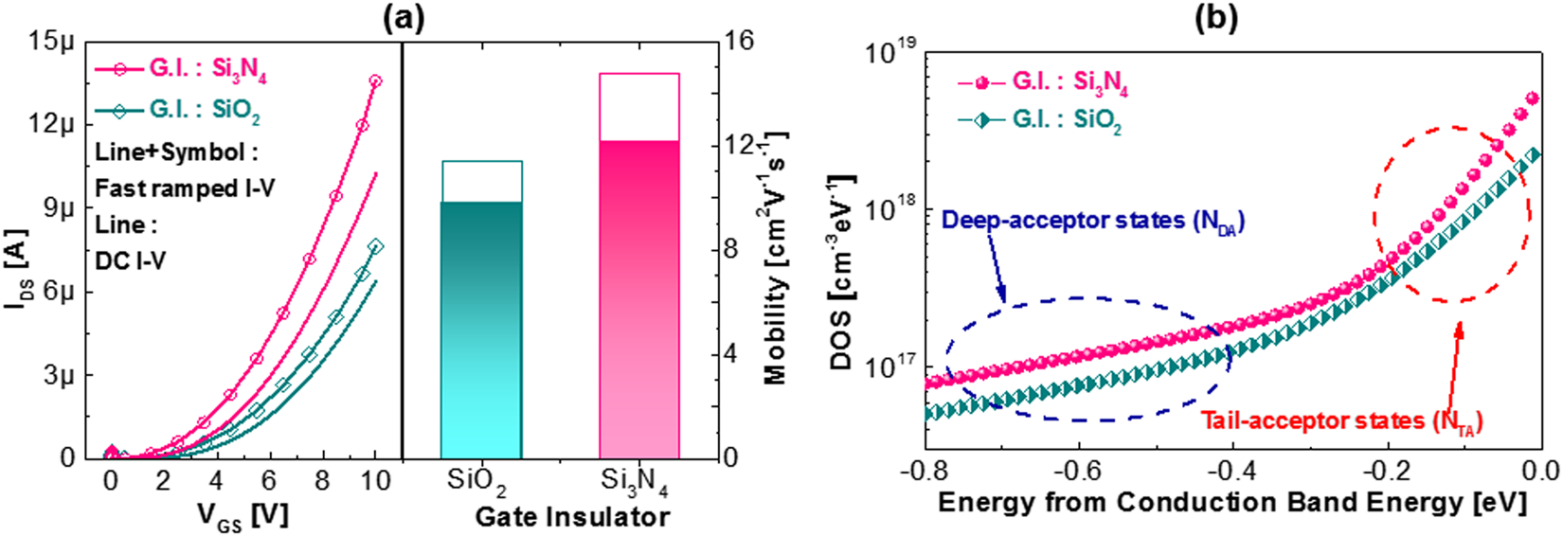
(**a**) Fast and DC *I-V* characteristics (left) and mobility (right) of *a*-IHZO TFTs with SiO_2_ and Si_3_N_4_ gate insulators. (**b**) Extracted subgap density of states versus energy characteristics of *a*-IHZO TFTs with SiO_2_ and Si_3_N_4_ gate insulators.

For a comparative study of the effect of defects on the charge trapping phenomena of oxide TFTs, we employed two different gate insulators, Si_3_N_4_ and SiO_2_, which significantly influence the interfacial and bulk quality owing to the strain effect, different atomic configuration, and hydrogen content. The left-hand graph of Fig. 3(a) shows the DC *I*–*V* and fast *I-V* (FIV) measurement data for the *a*-IHZO TFTs with Si_3_N_4_ and SiO_2_. The drain current levels and subthreshold slopes (S.S._fast_, _SiO2_ of 0.12 V/dec., S.S._DC_, _SiO2_ of 0.18 V/dec., S.S._fast_, _Si3N4_ of 0.13 V/dec., and S.S._DC_, _Si3N4_ of 0.20 V/dec.) were determined by transfer curve. Those measured by the FIV technique were better than those measured by the DC I-V technique. The S.S. value can be changed to the total trap density (N_tot_) using the following equation (1).

1 $${N}_{tot}=(\frac{SS\cdot \,\mathrm{log}(e)}{\frac{kT}{q}}-1)\frac{{C}_{ox}}{q}$$

Where *N*_*total*_ is the total trap density, *kT* is the thermal energy, *q* is the elementary charge and *C*_*ox*_ is the gate insulator capacitance. The *Ntot* values of IHZO with SiO_2_(Fast I-V), IHZO with SiO_2_(DC I-V), IHZO with Si_3_N_4_(Fast I-V) and IHZO with Si_3_N_4_(DC I-V) TFTs are 4.37 × 10^10^, 8.72 × 10^10^, 5.1 × 10^10^ and 1.02 × 10^11^ cm^−2^ ev^−1^, respectively. In addition, we added the values of subthreshold slope to the following sentence.

As presented in the right-hand graph of Fig. 3(a), the mobility values obtained by the FIV technique (*μ*_fast_, _Si3N4_ = 14.79 cm^2^/eV·s and *μ*_fast_, _SiO2_ = 12.1 cm^2^/eV·s) are, as expected, superior to those measured by the DC *I*–*V* technique (*μ*_DC_, _Si3N4_ = 11.42 cm^2^/eV·s and *μ*_DC_, _SiO2_ = 9.8 cm^2^/eV·s). The device in which Si_3_N_4_ is used as the gate insulating film shows higher drain current than that with SiO_2_ gate insulator. The use of a mixed gas of silane (SiH_4_) and ammonia (NH_3_) as a source gas during Si_3_N_4_ deposition leads to have a high concentration of hydrogen in Si_3_N_4_, acting as donor to the oxide semiconductor, shifting the threshold voltage of TFT toward negative gate bias direction due to high carrier concentration. In accordance with the percolation model, mobility increases in proportion to the carrier concentration^44,45^. Certainly, as shown in Fig. 3(a), Si_3_N_4_ devices exhibit high mobility. In agreement with earlier reports regarding metal oxide semiconductor field-effect transistors (MOSFETs), the DC *I*−*V* techniques used to extract various device parameters require comparatively long sweep/measurement times, such as a few seconds to tens of seconds, causing fast transient charge trapping and resulting in the underestimation of device performance^33–42^. Even for an *a*-IHZO TFT with SiO_2_ gate insulator, we observed significant differences in the value of drain current and mobility value measured by the DC *I*−*V* and FIV techniques. Moreover, the ratio of the mobilities, *μ*_fast_:*μ*_DC_, is higher for the *a*-IHZO TFT with Si_3_N_4_ gate insulator (29.5%) than for the *a*-IHZO TFT with SiO_2_ (23.5%), as presented in Fig. 3(a), indicating that the Si_3_N_4_ device is susceptible to fast charging. This was verified by the energy-dependent subgap DOS measured by MFM. The subgap DOS extraction of *a*-IHZO TFTs with two different gate insulators was begun by measuring the frequency-dependent capacitance−voltage (*C*−*V*) between source/drain electrodes and gate electrode, using an LCR meter^32^. Subsequently, we adopted the model (MFM) that was proposed by S. Lee and D. H. Kim and finally obtained a frequency-independent *C*−*V* and energy-dependent subgap DOS, as shown in Fig. 3(b) ^32^. The acceptor-like DOS of the *a*-IHZO TFT with Si_3_N_4_ gate insulator is higher than that of the *a*-IHZO TFT with SiO_2_ gate insulator. Table 1 summarizes the parameters of gate-insulator-dependent subgap DOS from the model *g*(*E*) = *N*_*TA*_·*exp*{(*E*-*E*_*C*_)/*kT*_*TA*_} + *N*_*DA*_·*exp*{(E-E_C_)/*kT*_*DA*_}, where *N*_TA_ is the acceptor like tail state density, *kT*_TA_ is the acceptor like tail state characteristic energy, *N*_DA_ is the acceptor like deep state density, and *kT*_DA_ is the acceptor like deep state characteristic energy.

**Table 1:** Summary of extracted parameters of density of states.

Gate Insulator	*N*_*TA*_ (cm^−3^eV^−1^)	*kT*_*TA*_ (eV)	*N*_*DA*_ (cm^−3^eV^−1^)	*kT*_*DA*_ (eV)
Si_3_N_4_	5.8 × 10^18^	0.06	3.9 × 10^17^	0.5
SiO_2_	2.3 × 10^18^	0.08	2.5 × 10^17^	0.5

To further understand the defects and transient charging mechanism, the *a*-IHZO TFTs were subjected to a constant bias, short pulse, and long pulse stresses under measurement temperatures ranging from 25 to 175 °C^43^. To observe the threshold voltage (*V*_*TH*_) shift, after a constant bias stress duration, the electrical stress was stopped and the *DC I-V* was measured^43^.

**Figure 4:**
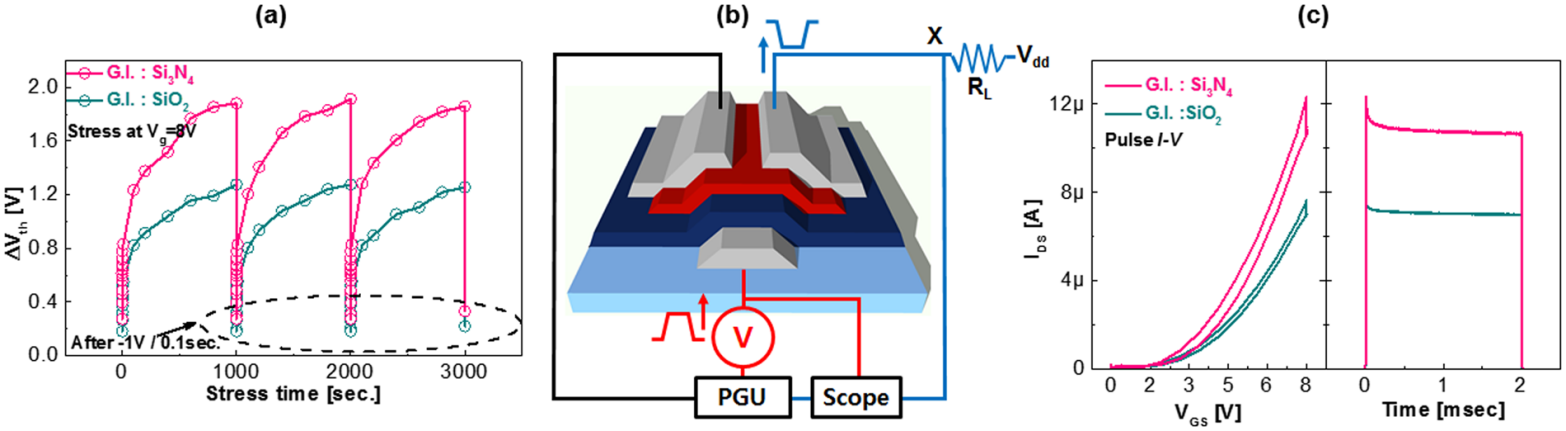
(**a**) Threshold voltage shift in *a*-IHZO TFTs during stress cycles, which include 1000 s at *V*_*GS*_ = 8 V followed by 0.1 s stress at the opposite bias of -1 V. (**b**) Schematic of pulse I-V measurement system (**c**) Pulse *I-V* characteristics (left) and corresponding transient current versus time characteristics (right) of *a*-IHZO TFTs with SiO_2_ and Si_3_N_4_ gate insulators.

Consistent with previous studies, we confirmed that under low bias stress, electron trapping shows a reversible phenomenon by applying de-trapping (opposite polarity) voltage^43^. As shown in Fig. 4(a), the *V*_*TH*_ value can be returned to the value before applying the stress voltage. Also, the dependence of the *V*_*TH*_ value under the bias stress (stress voltage of 8 V) hardly changes with each stress cycle. In addition, subthreshold slope (S.S. _SiO2_ of 0.18 V/dec., and S.S. _Si3N4_ of 0.20 V/dec.) is almost not affected by the bias stress. Thus, we believe that electron trapping takes place in pre-existing defects. Additionally, the low bias stress applied to the device does not generate a considerable number of defects. Figure 4(a) shows an initial sharp increase in *V*_*TH*_ for the first second of constant bias stress. To examine the initial charge trapping for the different quality of major charge transport layer, front channel (the channel near to the gate insulator) with relatively shallow energy, we performed a PIV technique on the *a*-IHZO TFTs with two different gate insulator materials such as Si_3_N_4_ and SiO_2_^33–42^. The PIV measurement schematics is presented in Fig. 4(b). A square wave pulse was applied to the gate electrode. The rise and fall times were both 10 µs and pulse width time was 2 ms. The gate voltage pulse was 8 V (Fig. 4(c)).

Figure 4(c) displays the PIV (left-hand graph) as well as transient current (right-hand graph) data. During the positive-bias pulse width, the degradation of the source to drain current (Δ*I*_*DS*_) results from the variation of threshold voltage (Δ*V*_*TH*_) owing to the trapped charge in the front channel Δ*I*_*DS*_ = (*W*/*L*)·*C*_*ox*_·*μ*·Δ*V*_*T*_, where *W* and *L* are the TFT channel width and length, respectively, *C*_*ox*_ is the gate oxide capacitance, and *μ* is the carrier mobility. According to the equation above, Δ*V*_*TH*_ can be calculated from Δ*I*_*DS*_:

2 $${\rm{\Delta }}{I}_{DS}=(\frac{{\rm{\Delta }}{I}_{DS}}{{I}_{DS}})\ast ({V}_{GS}-{V}_{TH}),$$

where Δ*I*_*DS*_ is the difference in the source to drain current between the end and start of the gate bias pulse, *I*_*DS*_ is the maximum source to drain current prior to charge trapping, *V*_*GS*_ is the gate voltage pulse amplitude, and *V*_*TH*_ is the threshold voltage.

**Figure 5:**
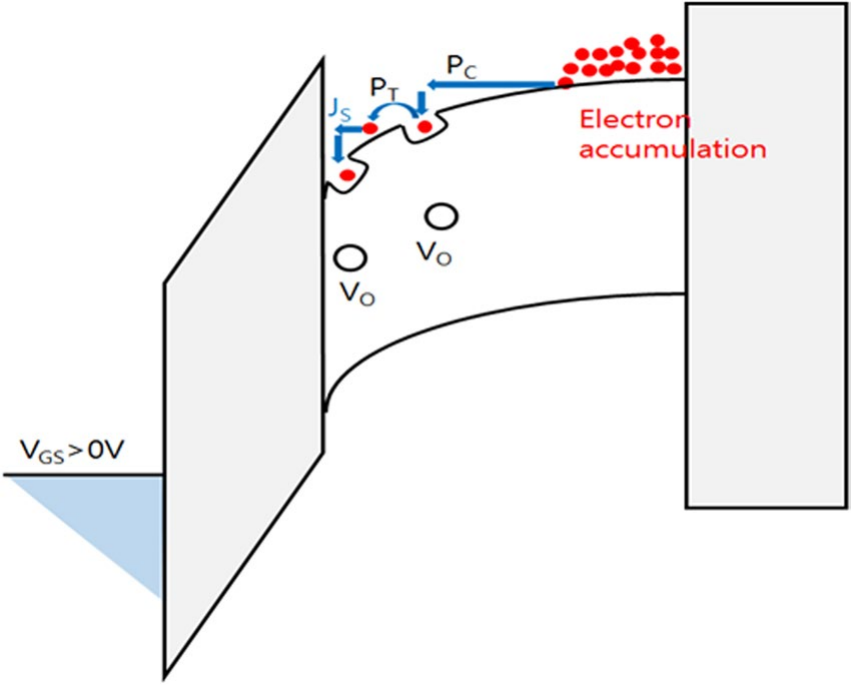
Schematic band diagram of *a*-IHZO TFTs with the proposed charge-trapping process model (*P*_*C*_: injected electrons from the conduction band and charge trapping at a shallow trap by drift process, *P*_*T*_: a temperature-activated charge de-trapping & re-trapping process).

The charge trapping in the pre-existing defects through several processes is described in Fig. 5. Electrons drift to the front channel and are charged in acceptor like defects [process *P*_*C*_ in Fig. 5]. Then, it follows temperature-dependent electron transfer between the defects (process *P*_*T*_). In oxide semiconductor channel, when acceptor-like defects are filled with electrons, they became electrically negative, which contributes to Δ*V*_*TH*_ and Δ*I*_*DS*_. As will be discussed below, a noticeable feature of acceptor-like defects in the front channel that contribute to fast and slow transient charging is that their energy states are located in a relatively shallow energy range of <0.4 eV below the conduction band minimum. The charging process *P*_*C*_ is expected to have a short charging characteristic time because of the very low trap energy and high DOS in the *a*-IHZO conduction band. Thus, the charging process *P*_*C*_ is the major constituent of fast transient charging. Here, we concentrate on fast transient charge trapping induced by *P*_*C*_ (fast component) and slow transient charge trapping caused by temperature-activated electron transport, *P*_*T*_ (slow component). At the beginning of the constant bias stress, the initial (~100 μs) increase in Δ*V*_*TH*_ (see, for example, Fig. 4(a)) is comparable to the Δ*V*_*TH*_ obtained by the PIV measurement method in the μs range, while during the subsequent bias stress of several seconds, Δ*V*_*TH*_ gradually increases with stress time, contributing to the observed stress-time-dependent Δ*V*_*TH*_. This indicates that there are two charging processes with different charging characteristic time. We found that fast transient charging (*P*_*C*_) occurs in the μs range and slow transient charging (temperature-dependent charging, *P*_*T*_) starts to take place after a few seconds. Thus, fast transient charging (*P*_*C*_) is significantly responsible for the initial Δ*V*_*TH*_ (Fig. 4(a)). Different time scales, such as 10^−6^–10^−4^ s (fast component) and 10–10^3^ s (slow component), allow the categorization of fast and slow processes, as described in the model.

### Fast transient charging

To investigate fast and slow charging, we applied a simplified model proposed by G. Bersuker^43^. In this model, we assume that channel electrons drift to the defects and can be negatively charged when they occupy defects whose energies are in quasi-resonance. Following the assumption above, equation (3) shows the kinetics of fast charging. And the solution of this equation can be represented by Eq. (4):

3 $$\frac{\partial n}{\partial t}=p({N}_{O}-n),$$

4 $${\rm{n}}={N}_{O}(1-{e}^{-pt}),$$

Where, *n* is the density of the occupied traps, *t* is the time, *p* is the probability of a electron-trapping event, *N*_0_ is the total density of available traps.

**Figure 6:**
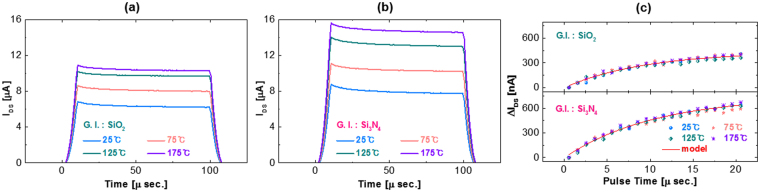
Source to drain current of the *a*-IHZO TFTs with (**a**) SiO_2_ and (**b**) Si_3_N_4_ gate insulators during the pulse width (100 µs) at the gate pulse *V*_*GS*_ of 8 V measured at various temperatures. (**c**) Source to drain current shift during the initial 20 µs of the pulse in Fig. 5(a) and (b).

To evaluate the influence of temperature on fast transient charging, the transient current versus time characteristics of the *a*-IHZO TFTs with SiO_2_ and Si_3_N_4_ gate insulators were measured by applying a short pulse (100 µs) at 25 to 125 °C (Fig. 6(a) and (b)). As such in previous study^46^, as seen in Figs 6(a) and 5(b), the source to drain current tends to increase with increasing measurement temperature. According to the model, more electrons escape from the localized state at relatively high measurement temperature^46^. On the other hand, Δ*I*_*DS*_ for the initial 20 μs of the pulse width is almost the same regardless of measurement temperature, as seen in Fig. 6(c). A representative example of fitting Equation (4) to the measured source to drain current with time for Si_3_N_4_ and SiO_2_ gate-insulator-stacked *a*-IHZO devices is presented in Fig. 6(c), where the fitting was performed to Δ*I*_*DS*_ for the initial 20 μs of the pulse width in Fig. 6(a) and (b), which is much shorter than the time it takes for the Δ*I*_*DS*_ to saturate. We should notice that time dependence of the drain current does not depend on temperature, representing that fast charging is not a temperature-activated process. The obtained *N*_0_ is of the order of 10^13^ cm^−2^ (Si_3_N_4_ gate insulator) and 10^12^ cm^−2^ (SiO_2_ gate insulator). Coulomb repulsion, which prevents charge trapping at most available defect sites, acts between trapped charges, the final density seems to be low.

### Slow transient charging

It has been demonstrated in previous studies that slow transient charging is as effectively de-trapped as fast transient charging, under the same detrapping voltage^47,48^. This shows that traps with fast and slow transient charging have similar detrapping kinetics, suggesting that the traps of the two types of charging have similar physical characteristics. The model we apply and study begins with the assumption that the fast and slow transient charging occur in the same traps^43^.

In accordance with the slow transient charging model, slow transient charging might be attributed to the capture of secondary electrons, which stem from the traps, charged by the fast drift process (*P*_*C*_) (Fig. 5)^43^. Thus, when some trapped electrons are activated from the traps by thermal energy and are hopping over the conduction band minima, they can be re-trapped in the surrounding empty traps before these electrons gain sufficient kinetic energy. This is the trap-conduction band-trap process (*P*_*T*_) (Fig. 5).

The fast (microsecond scale) and slow (second scale) transient charging have a very large difference in charging characteristic time scale, so a fast drift charging process occurs immediately in the traps where thermally activated detrapping occurs and this continuously provides activated electrons to continue the slow transient charging.

The slow transient charging process can be expressed in the form Eq. (5)

5 $${\rm{N}}={N}_{S}\sum (1-{e}^{-{p}_{s}(i)t}),$$

where *N* is the trap density, *N*_*S*_ is the density of the secondary traps positioned close the filled traps, and *p*_*s*_(*i*) = *σ*_*s*_ *J*_*s*_(*i*), where *σ*_*s*_ is the capture cross-section for the electrons, and *J*_*s*_ expressed in Eq. (6) is defined by electrons that are thermally activated and emitted from the traps (Fig. 5).

6 $${J}_{s}(i)=n\frac{1}{\tau }\exp (-\frac{{E}_{i}}{kT}),$$

where *J* is the current density, *n* is the density of the occupied fast traps, *τ* is the de-trapping time constant, *E*_*i*_ is the trap energy, *k* is the Boltzmann constant, and *T* is the temperature.

**Figure 7:**
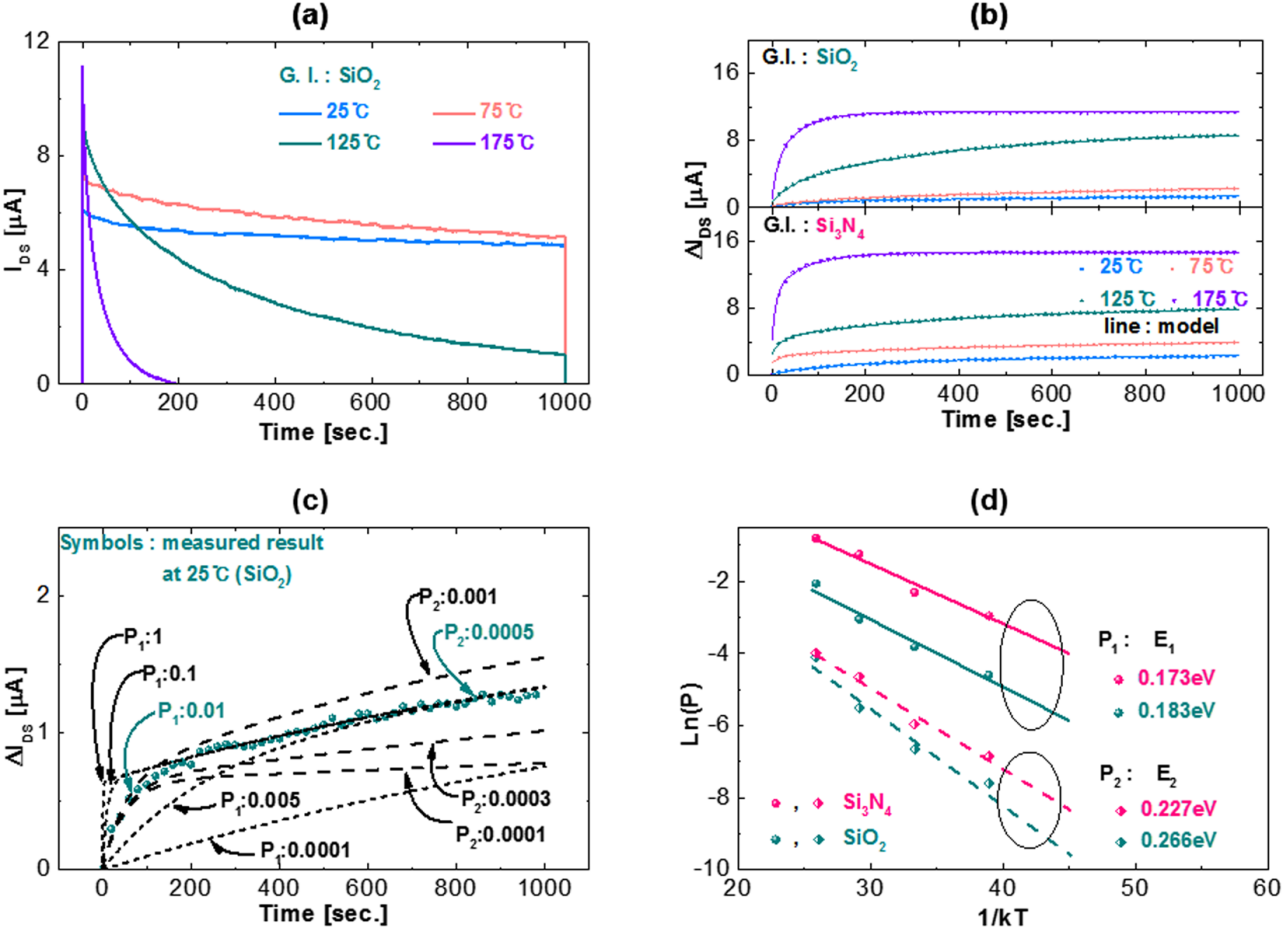
(**a**) Source to drain current of the *a*-IHZO TFTs with SiO_2_ gate insulator during the huge long pulse width (1000 s) at the gate pulse *V*_*GS*_ of 8 V measured at various temperatures. (**b**) Source to drain current shift of the *a*-IHZO TFTs with SiO_2_ and Si_3_N_4_ gate insulators during the huge long pulse width (1000 s) of the pulse in Fig. 6(a). (**c**) Source to drain current shift with different *P*_*i*_ during the huge long pulse width (1000 s), measured at 25 °C, of the pulse in Fig. 6(a). (**d**) Extracted activation energies E_i_ corresponding to P_i_ data of *a*-IHZO TFTs with SiO_2_ and Si_3_N_4_ gate insulators.

The transient current was measured by applying a long pulse stress (pulse width: 1000 sec.) to the *a*-IHZO TFTs with SiO_2_ and Si_3_N_4_ gate insulators at a temperature from 25 to 125 °C to verify the temperature dependent slow transient charging model described above. Representatively, the measured data for the *a*-IHZO TFT with SiO_2_ device is shown in Fig. 7(a). The results of fitting the Δ*V*_*TH*_ values for the whole temperature range using Eq. (5) are shown in Fig. 7(b). The fitting was repeatedly carried out by increasing the terms one by one, starting with a single term in the sum of Eq. (5). Fitting the Eq. (5) in Fig. 7(b) requires two terms, which implies that slow charging occurs in more than one type of defects. The fitting of equation (6) for all temperature data requires two terms, *i* = 1, 2. The fitting process to obtain parameters *p*_*i*_(*T*) and *N*_*s*_ was repeated for various stress temperatures (25, 75, 125, and 175 °C) (Fig. 5(b)). The measured Δ*I*_*DS*_ data of 25 °C and the theoretical line of equation (5) are plotted with different p_i_ (Fig. 7(c)). Among various fitting values, the measurement data can best be described as *p*_1_ is 0.01 and *p*_2_ is 0.0005. The slope of [ln(*p*_*i*_) *vs* 1/*kT*] presents the energy barriers height *E*_*i*_ for the detrapping electrons influencing on the charge flux *J*_*s*_(*i*) (*i* = 1, 2) in Eq. (6): *E*_1_ = 0.17 eV, *E*_2_ = 0.23 eV (Si_3_N_4_ gate insulator), and *E*_1_ = 0.18 eV, *E*_2_ = 0.27 eV (SiO_2_ gate insulator) (Fig. 7(d)). The available trap density (*N*_*S*_) which can recapture the secondary electrons, are of the order of 10^9^ cm^−2^ (Si_3_N_4_ gate insulator) and 10^8^ cm^−2^ (SiO_2_ gate insulator), implying that most of the secondary electrons from the initial traps are not re-trapping. The *N*_0_ and *N*_*s*_ values of the *a*-IHZO TFT with Si_3_N_4_ gate insulator are each approximately one order of magnitude higher than those of the *a*-IHZO TFT with SiO_2_ gate insulator.

## Discussion

In this study, we proposed a quantitative analysis of the dominant mechanism of device instability stemming from the charge trapping behavior and defect characteristics of oxide TFTs. To systematically analyze the charge trapping mechanism, we probed the subgap DOS by MFM measurements, as well as examining fast/slow transient charging and trap energy by short pulse stress and long pulse stress. These analyses provide a foundation for an instability model of oxide TFTs.

The obtained results show that the *a*-IHZO TFT with Si_3_N_4_ gate insulator is vulnerable to fast/slow charge trapping, causing significant device instability, high acceptor-like defect density, and shallow trap energy compared with those of the *a*-IHZO TFT with SiO_2_ gate insulator. We believe that this method will help to understand the defects of oxide semiconductor transistor and will guide the direction of defect control.

## Methods

### Device Fabrication

Molybdenum metal used as a gate/source/drain electrode was deposited by DC sputtering. For the preparation of both the Si_3_N_4_ and SiO_2_ gate insulators, we used plasma-enhanced chemical vapor deposition (PECVD) in the gas chemistry of SiH_4_/NH_3_ and SiH_4_/N_2_O, respectively. Then, *a*-IHZO oxide semiconductor films of thickness 40 nm were deposited by means of a radio-frequency plasma sputtering method. The IHZO targets were composed of a mixture of HfO_2_, In_2_O_3_, and ZnO powders. For example, the IHZO (7 mol% - Hf content) target was composed of HfO_2_: In_2_O_3_: ZnO = 0.07: 1: 1. The *a*-IHZO (7 mol% - Hf content) film composition was verified by analyzing the energy dispersion spectrometry (see the Fig. 1(c)). For the comparison purpose of effect of IHZO semiconductor regarding Hf contents, the Hf content of the IHZO targets were prepared in contents of 4, 7, and 10 mol%. After the formation of the *a*-IHZO layer, the etch stopper layer was formed of SiO_2_ using PECVD. Subsequently, the TFT devices were passivated with PECVD SiO_2_ followed by contact etching.

### Device Characterization

The DC *I*–*V* measurements were performed using a semiconductor parameter analyzer (Agilent 4156 C), and the fast *I*–*V* (FIV) and pulse *I*–*V* (PIV) measurements were carried out using a pulse-generation unit (Agilent B1104A) and a digital oscilloscope (Agilent MSO6052A). For the DC *I*–*V* measurements, the voltage sweep rate was 1 V/s, whereas for the FIV and PIV measurements, the voltage scan rate was 1 V/µs. The multi-frequency measurements (MFM) were performed using an LCR meter (Agilent 4284 A).
